# Spatial Evaluation and Modeling of Dengue Seroprevalence and Vector Density in Rio de Janeiro, Brazil

**DOI:** 10.1371/journal.pntd.0000545

**Published:** 2009-11-10

**Authors:** Nildimar Alves Honório, Rita Maria Ribeiro Nogueira, Cláudia Torres Codeço, Marilia Sá Carvalho, Oswaldo Gonçalves Cruz, Mônica de Avelar Figueiredo Mafra Magalhães, Josélio Maria Galvão de Araújo, Eliane Saraiva Machado de Araújo, Marcelo Quintela Gomes, Luciane Silva Pinheiro, Célio da Silva Pinel, Ricardo Lourenço-de-Oliveira

**Affiliations:** 1 Laboratório de Transmissores de Hematozoários, Instituto Oswaldo Cruz, Fiocruz, Rio de Janeiro, Brasil; 2 Laboratório de Flavivírus, Instituto Oswaldo Cruz, Fiocruz, Rio de Janeiro, Brasil; 3 Programa de Computação Científica Fiocruz-PROCC, Instituto Oswaldo Cruz, Fiocruz, Rio de Janeiro, Brasil; 4 Centro de Informação Científica e Tecnológica, Laboratório de Processamento de Imagens Fiocruz-ICICT, Rio de Janeiro, Brasil; 5 União Ativista Defensora do Meio Ambiente-UADEMA/NAPVE/FIOCRUZ, Rio de Janeiro, Brasil; Duke University-National University of Singapore, Singapore

## Abstract

**Background:**

Rio de Janeiro, Brazil, experienced a severe dengue fever epidemic in 2008. This was the worst epidemic ever, characterized by a sharp increase in case-fatality rate, mainly among younger individuals. A combination of factors, such as climate, mosquito abundance, buildup of the susceptible population, or viral evolution, could explain the severity of this epidemic. The main objective of this study is to model the spatial patterns of dengue seroprevalence in three neighborhoods with different socioeconomic profiles in Rio de Janeiro. As blood sampling coincided with the peak of dengue transmission, we were also able to identify recent dengue infections and visually relate them to *Aedes aegypti* spatial distribution abundance. We analyzed individual and spatial factors associated with seroprevalence using Generalized Additive Model (GAM).

**Methodology/Principal Findings:**

Three neighborhoods were investigated: a central urban neighborhood, and two isolated areas characterized as a slum and a suburban area. Weekly mosquito collections started in September 2006 and continued until March 2008. In each study area, 40 adult traps and 40 egg traps were installed in a random sample of premises, and two infestation indexes calculated: mean adult density and mean egg density. Sera from individuals living in the three neighborhoods were collected before the 2008 epidemic (July through November 2007) and during the epidemic (February through April 2008). Sera were tested for DENV-reactive IgM, IgG, Nested RT-PCR, and Real Time RT-PCR. From the before–after epidemics paired data, we described seroprevalence, recent dengue infections (asymptomatic or not), and seroconversion. Recent dengue infection varied from 1.3% to 14.1% among study areas. The highest IgM seropositivity occurred in the slum, where mosquito abundance was the lowest, but household conditions were the best for promoting contact between hosts and vectors. By fitting spatial GAM we found dengue seroprevalence hotspots located at the entrances of the two isolated communities, which are commercial activity areas with high human movement. No association between recent dengue infection and household's high mosquito abundance was observed in this sample.

**Conclusions/Significance:**

This study contributes to better understanding the dynamics of dengue in Rio de Janeiro by assessing the relationship between dengue seroprevalence, recent dengue infection, and vector density. In conclusion, the variation in spatial seroprevalence patterns inside the neighborhoods, with significantly higher risk patches close to the areas with large human movement, suggests that humans may be responsible for virus inflow to small neighborhoods in Rio de Janeiro. Surveillance guidelines should be further discussed, considering these findings, particularly the spatial patterns for both human and mosquito populations.

## Author Summary

Dengue is a major public health problem in many tropical regions of the world, including Brazil, where *Aedes aegypti* is the main vector. We present a household study that combines data on dengue fever seroprevalence, recent dengue infection, and vector density, in three neighborhoods of Rio de Janeiro, Brazil, during its most devastating dengue epidemic to date. This integrated entomological–serological survey showed evidence of silent transmission even during a severe epidemic. Also, past exposure to dengue virus was highly associated with age and living in areas of high movement of individuals and social/commercial activity. No association was observed between household infestation index and risk of dengue infection in these areas. Our findings are discussed in the light of current theories regarding transmission thresholds and relative role of mosquitoes and humans as vectors of dengue viruses.

## Introduction

Dengue is a mosquito-borne viral infection, considered a major public health problem in many tropical regions of the world, including Brazil [1],[2]. *Aedes aegypti* is the most important dengue vector worldwide [3]–[5] and the only known vector in Brazil [6]. Dengue infection can manifest itself as clinically unapparent, an undifferentiated febrile illness, classic dengue fever (DF), or dengue hemorrhagic fever (DHF). Prevalence of dengue is highest in tropical areas of Asia and the Americas, with 50–100 million estimated cases of dengue fever and 250,000–500,000 cases of dengue hemorrhagic fever occurring annually worldwide as explosive outbreaks in urban areas [7],[8].

**Figure 1:**
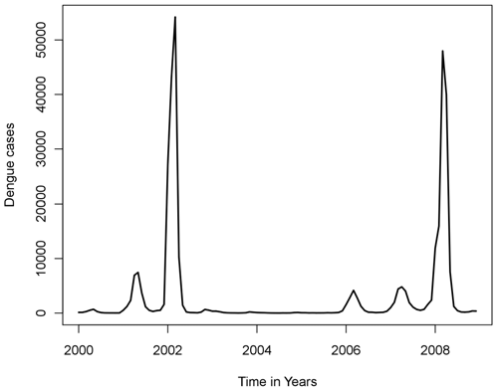
Notified dengue cases in Rio de Janeiro State from 2000 to 2008. Monthly cases showing two large epidemic in 2002 and 2008 and three small outbreaks in 2001, 2006 and 2007 (Source: State Secretary of Healthy of Rio de Janeiro 2008).

In Brazil, three dengue virus serotypes (DENV) have been introduced through Rio de Janeiro in the past three decades: DENV-1 in 1986 [9], DENV-2 in 1990 [10], and DENV-3 in 2000 [11]. Figure 1 shows the time series of dengue cases in Rio de Janeiro State from 2000 to 2008 [12]. The introduction of DENV-3 in the state of Rio de Janeiro led to severe epidemics in 2002 with the largest number of cases (288,245 notified), with 1,831 DHF cases and 91 deaths, corresponding to 1,735 reported cases per 100,000 inhabitants [13], and a case-fatality ratio of 3.15∶10,000. Eight years later, in 2007–2008, during the current study, Rio de Janeiro (and Brazil) experienced the most severe dengue epidemics ever reported in terms of morbidity and mortality [14]. During this period, 322,371 cases and 240 deaths were registered, with 100 deaths due to DHF/dengue shock syndrome (DSS) and 140 due to other dengue-related complications [12]. That represented a case-fatality rate of 9.4∶10,000. Contrasting with the previous epidemics, the 2008 epidemic, essentially caused by DENV-2, was characterized by a higher incidence of severe cases in children. In fact, 36% of deaths reported occurred in individuals ≤15 years old [12],[15].

Rio de Janeiro presents highly favorable conditions for transmission of dengue [13], as shown by serological cross-sectional surveys carried out after the arrival of DENV-1 and DENV-2. In 1987, after the first wave, 45.5% of schoolchildren were positive for DENV-1 haemagglutination inhibition antibodies (HAI) [16]. HAI antibody persists for a long period, but is highly cross-reactive [3]. In the neighbor city of Niterói, 55% of schoolchildren were positive in 1988, and 66% in 1992 (after the arrival of DENV-2) [17],[18]. In Paracambi, another neighbor city, 29.2% schoolchildren were positive in 1997 [19].

Dengue surveillance and control in large urban areas with high levels of dengue transmission pose important challenges. Clinical surveillance is impaired by the high proportion of asymptomatic infections [20],[21],[22], and mosquito surveillance is very time and resource consuming. Moreover, despite the theoretical association between vector abundance and risk of transmission, the quantitative nature of this relationship is poorly known [23]. Understanding the epidemiology of this disease requires studies that integrate epidemiological and entomological data [19],[21],[24],[25].

The main objective of this study is to model the spatial patterns of seroprevalence in three neighborhoods with different socioeconomic profiles in Rio de Janeiro. As blood sampling coincided with the peak of dengue transmission, we were also able to identify recent dengue infections and visually relate them to *Aedes aegypti* spatial distribution abundance. We analyzed individual and spatial factors associated with seroprevalence using Generalized Additive Model (GAM).

## Methods

### Study sites

**Figure 2:**
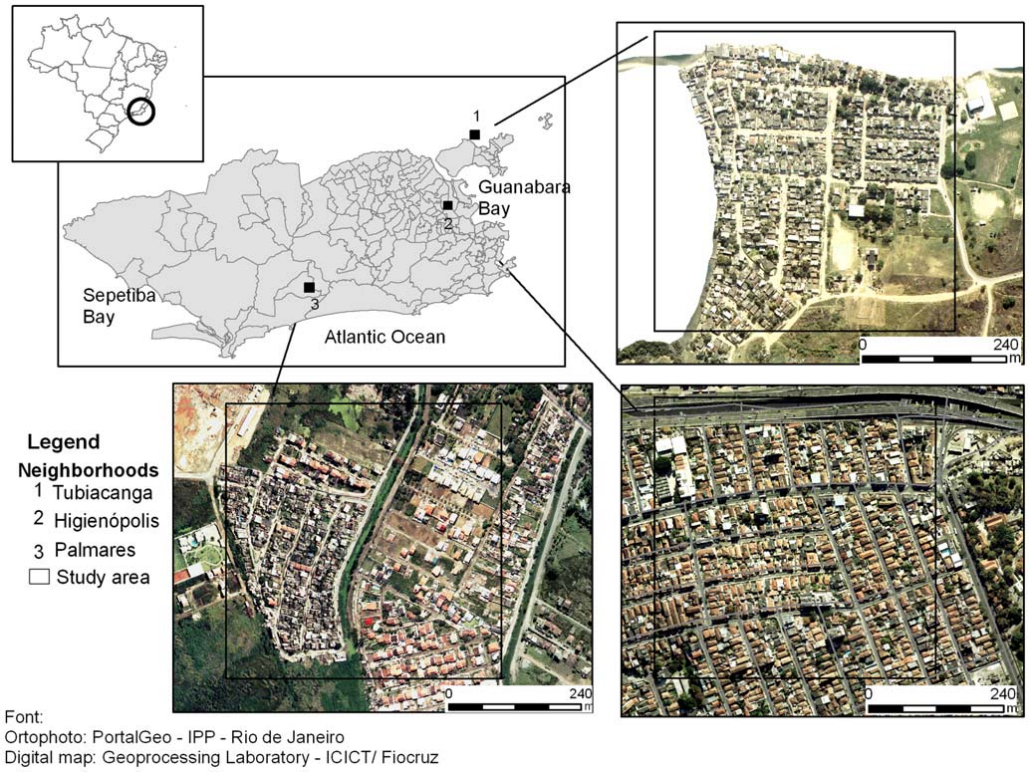
Map of Rio de Janeiro showing the location of the three study sites. Tubiacanga (1) is a suburban mostly a residential neighborhood, located in an island, in the Guanabara Bay. Access is limited to a single main route. Higienópolis (2) is an urban neighborhood densely populated, totally urbanized: paved streets, with adequate water supply and garbage collection services. However it is surrounded by some of the largest slums of the city. Palmares (3) is a recently settled slum between a rain forest mountainous range and a polluted river, with just one road access.

Surveys were performed in three neighborhoods of Rio de Janeiro city: Higienópolis, Tubiacanga, and Palmares, which differ in human population density, sanitation, vegetation cover, and history of dengue (Fig. 2). Since neighborhoods were large and heterogeneous, we restricted the survey to an area of approximate 0.25 km^2^ in each one [26].

#### Higienópolis, the urban area (22°52′25″ S; 43°15′41″ W)

Higienópolis is an urban neighborhood located within a densely populated area of Rio de Janeiro city (15,891 inhabitants/km^2^). In the selected area, approximately 4,076 people live in 1,308 houses [27]. The Yellow Line, one of the city's busiest major highways, crosses and connects Higienópolis to numerous suburban localities. The area is totally urbanized: streets are paved, with adequate water supply and garbage collection services. Residents, mostly middle class and elderly, live in one story houses with no yard or small cemented/paved ones. Higienópolis, however, is surrounded by some of the largest slums of Rio de Janeiro, characterized by poor infrastructure normally associated with favorable conditions for *Ae. aegypti* proliferation.

#### Tubiacanga, the suburban area (22°47′08″ S; 43°13′36″ W)

Tubiacanga is an isolated suburban neighborhood at the tip of the Governador Island, in the Guanabara Bay. Access to the area is limited to a single main route, a 2.1 km paved road, which connects the area to the nearest neighborhood. Tubiacanga is mostly a residential neighborhood, with 2,521 individuals living in 682 one story houses – with large outdoors yards - in an area of ca. 0.25 km^2^ [27]. Streets are unpaved, garbage collection is regular, but access to water is irregular, and residents often store water in containers, which are potential development sites for immature *Ae. aegypti* [28],[29].

#### Palmares, the suburban slum (22°59′26″ S; 43°27′36″ W)

Palmares is a recently settled slum between a rain forest mountainous range and a polluted river, located at one of the major axes of the city's expansion. Palmares' population density is 2,733 inhabitants/km^2^. In the selected area of 0.25 km^2^ ca. 1749 people live in 498 houses [27]. Housing distribution is crowded and irregular, with narrow unpaved alleys. Palmares is a relatively isolated area, with just one road access.

### Study design

The serological surveys were carried out in July-November 2007 and February-April 2008, the latter coinciding with the 2008 high transmission period [12]. The study areas had been under entomological surveillance since September 2006 (see Mosquitoes surveillance section) [26]. The entomological surveillance consisted of weekly collections of *Ae. aegypti* eggs and adults using traps located in 80 households per site. All householders participating in the entomological surveillance were invited to participate in the serological surveys. Only 72 out of 240 householders agreed to participate (13 in Higienópolis, 31 in Tubiacanga and 28 in Palmares). To increase the sample, we invited additional residents from nearby houses, reaching a total of 171 participating households (19 in Higienópolis, 93 in Tubiacanga and 59 in Palmares), with 337 individuals (44 in Higienópolis, 162 in Tubiacanga and 131 in Palmares). Since previous studies reported lower seropositive rates in the younger age classes [16],[18],[19], we concentrated our sample effort in the age group of 1–20 years old to increase the chance of detecting seroconversion events [30]. However, due to problems related to participant refusal, particularly for small children in the urban area, older people were included as well, to increase the sample size. The range and median age in the sample is presented in Table 1. A questionnaire was applied to each enrolled individual, with questions regarding sex, age, education level, yellow fever (YF) vaccination status, clinical symptoms of dengue-like disease and past dengue episodes. The location of each household was determined by a hand-held, 12 channel global positioning system (Garmin), which accurate to 15 m.

**Table 1:** Serological surveys, July-November 2007 and February-April 2008 in three areas, Rio de Janeiro, Brazil.

Area (type)		Serum sample (IgM) - Surveys			Serum sample (IgG) - Surveys		
		1^st^	2nd	Seroconversion paired sample (IgM)	1st	2nd	Seroconversion paired sample (IgG)
Higienópolis (Urban)	*n*	43	29	28	43	29	28
	Positives	1*	4**	2	37	27	1
	Median Age (range)	28.5 (4–79)	42 (4–79)	40.50 (4–79)	28.5 (4–79)	42 (4–79)	40.50 (4–79)
Tubiacanga (Suburban)	*n*	157	122	117	157	122	117
	positives	2^+^	5^++^	4	90	75	4
	Median Age (range)	11 (4–74)	12 (4–74)	12 (4–74)	11 (4–74)	12 (4–74)	12 (4–74)
Palmares (Suburban slum)	*n*	126	107	102	126	107	102
	positives	6^†^	15^††^	11	72	73	8
	Median Age (range)	11 (1–52)	11 (1–52)	10 (1–52)	11 (1–52)	11 (1–52)	10 (1–52)
**Total**	**positives/total**	**9/326**	**24/258**	**17/247**	**199/326**	**175/258**	**13/247**

Number of asymptomatic individuals: *1; **2; ^+^ 2; ^+ +^ 4; ^†^ 4; ^††^ 10.

### Case definition

*Recent dengue infection* was defined by the detection of DENV IgM antibodies in any sample (first or second sample) within the last 6 weeks or so. *Seroprevalence* was defined by detection of DENV IgG antibodies in the first sample (July–November/2007). *Seroconversion* was defined only for the paired samples – negative in the first sample and positive in the second one – considering both IgM and IgG. *Primary infection* was defined as a negative IgG in the first sample with positive IgM in the second and *secondary infection* when DENV IgG antibodies were detected in the first sample. Individuals with DENV IgM antibodies were considered *asymptomatic cases* when clinical definition of dengue – high fever, accompanied by at least two of the associated symptoms: headache, myalgia, arthralgia retro-orbital pain and rash – was not met [31].

### Blood sample collections

A blood sample (5 mL) was collected from all participants during the household visit, stored at −20°C and processed within 12 hours. Sera were tested for DENV- reactive IgM and IgG immunoglobulin by using PANBIO dengue IgM capture and dengue IgG indirect Elisa (Brisbane, Australia).

### RNA extraction

Viral RNA for the nested RT-PCR and real-time RT-PCR assays was extracted from 140 µL of serum samples by the QIAamp Viral RNA Mini Kit (QIAGEN, Valencia, CA), according to the manufacturer's instructions. RNA was eluted in 60 µL of buffer (AVE) and stored at −70°C. For the quantitative TaqMan assay, a 10-fold-dilution series containing a known amount of target viral RNA (10^7^ RNA copies/mL) was used for RNA extraction.

### Nested reverse transcriptase PCR assay

The nested RT-PCR protocol for DENV detection and typing was performed on serum samples, which tested DENV IgM positive according to [32].

### Real-time reverse transcriptase PCR (TaqMan) assay

One-step real-time RT-PCR assays were performed in the ABI Prism 7000 Sequence Detection System (Applied Biosystems, Foster City, CA) in all IgM positive samples. Briefly, samples were assayed in a 25 µl reaction mixture containing 5 µl of extracted RNA, 1 µl of 40X Multiscribe enzyme plus RNAse inhibitor, 12.5 µl TaqMan 2X Universal PCR Master Mix (Applied Biosystems, Foster City, CA) and 300 nM of each specific primer and fluorogenic probe. Positive and negative controls were included. To detect specific DENV1-2, primer and probe sequences were obtained from [33]. To detect specific DENV-3, primer and probe sequences were obtained from [34]. The TaqMan probe was labeled at the 5′ end with the 5-carboxyfluorescein (FAM) reporter dye and at the 3′ end with 6-carboxy-*N,N,N′,N′*-tetramethylrhodamine (TAMRA) quencher fluorophore. The number of viral RNA copies detected was calculated by generating a standard curve from 10-fold-dilutions of DENV-3 RNA, isolated from a known amount of local virus propagated in *Aedes albopictus* C6/36 cells [13], the titer of which was determine by plaque assay. The same model of DENV-3 standard curve was applied to build DENV-1 and DENV-2 curves. Quantitative interpretation of the results obtained was performed by interpolation from the standard curve included in each independent run for each serotypes.

### Mosquito surveillance

Entomological surveillance was carried out with two types of traps for ovipositing females, egg traps and adult traps. Egg traps are black plastic containers, filled with 300 ml of a 10% hay infusion, and a wooden paddle held on the wall for oviposition [26],[35],[36],[37]. Adult traps (version 1.0, Ecovec Ltd) consists of a matte black container (16 cm high×11 cm diameter) with approximately 280 ml of water and a removable sticky card. A synthetic oviposition attractant was used to attract gravid female mosquitoes [38]. Surveillance was conducted weekly from September, the 6^th^ 2006 to March, 24^th^ 2008 in the three study areas, encompassing two wet-hot seasons and one dry-cool season. In each study area, 40 adult traps and 40 egg traps were installed in a random sample of premises [26]. Two infestation indexes were calculated: mean adult density (MAD  =  number of trapped female *Ae. aegypti*/number of adult traps and mean egg density (MED  =  number of collected eggs/number egg traps). Details on the entomological methods and results are described in [26]. To evaluate potential heterogeneities in the spatial distribution of mosquito abundance during the serological surveys, we aggregated the weekly entomological collections over time, from April/2007 to March/2008, into a single index. Recent dengue infections are plotted on this vector abundance map to inspect for possible associations. Breteau Index (number of *Ae. aegypti*-positive containers per 100 houses) measured in March, June, August, November of 2007 and January and April of 2008 in each study area was also obtained from Public Health Office of Rio de Janeiro city.

### Data analysis

The number of r*ecent dengue infections* was very small, and consequently, not statistically modeled (descriptive data in Table 1). To compare and possibly to advance further investigations, the coordinates of negative and positive (in any sample) DENV IgM antibodies were mapped over the aggregated distribution of adult mosquito abundance. The technique to build the interpolated surface is presented in the section below.

To compare *seroprevalence* among the areas we standardized the proportion of positive samples (direct method) using the total number of samples in all areas. S*eroprevalence* data was analyzed using a Generalized Additive Model (GAM): a statistical model that extends the generalized linear models to include non-parametric smoothing terms. In the generalized linear model, the response variable belongs to the exponential family, and its mean value is related to the linear predictors through a link function. The canonical link function for binomial response, such as positive or negative sera, is the logit link. To evaluate possible non-linearity of the age effect on the outcome we used a smooth-spline and plotted the predicted against the observed value. The spatial distribution was modeled using a bi-dimensional smooth function [39]. The complete model thus included a set of directly observed covariates and a function – in our case, a thin plate spline – applied on the geographical coordinates of each household, as depicted in the equation below:
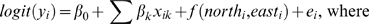


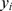
 is the response variable, 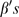 are the slope coefficients of the model, so 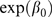 is the adjusted odds ratio, 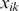 are the explanatory variables at the individual and household levels, the function 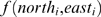 is a smooth function of geographic co-ordinates and 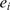 are the residuals. All covariates with a p-value ≤0.10 in the univariate analysis were included in the multivariate model. The approach used to analyze the spatial distribution started with a model with just the smooth function of the coordinates. Then explanatory variables were included successively until the final adjusted model was obtained. Contour lines at p-value ≤0.05 were drawn on the maps to identify areas with significantly higher (red lines) and lower risk (blue lines) than the overall mean.

In the case of the mosquito interpolation surface, the adults counts were the outcome variable and the smoothed geographic coordinates of the adult traps were the independent variables. All statistical analyses were performed using the statistical software R 2.8.1 [40], with library mcgv [41].

### Ethical considerations

Ethical clearance was obtained from the Ethical Committee in Research (CEP 365/07) from the Oswaldo Cruz Foundation, Ministry of Health, Brazil. Written consent to participate in the two surveys was obtained from each participant and in case of minor, from their legal guardians.

## Results

### General

**Figure 3:**
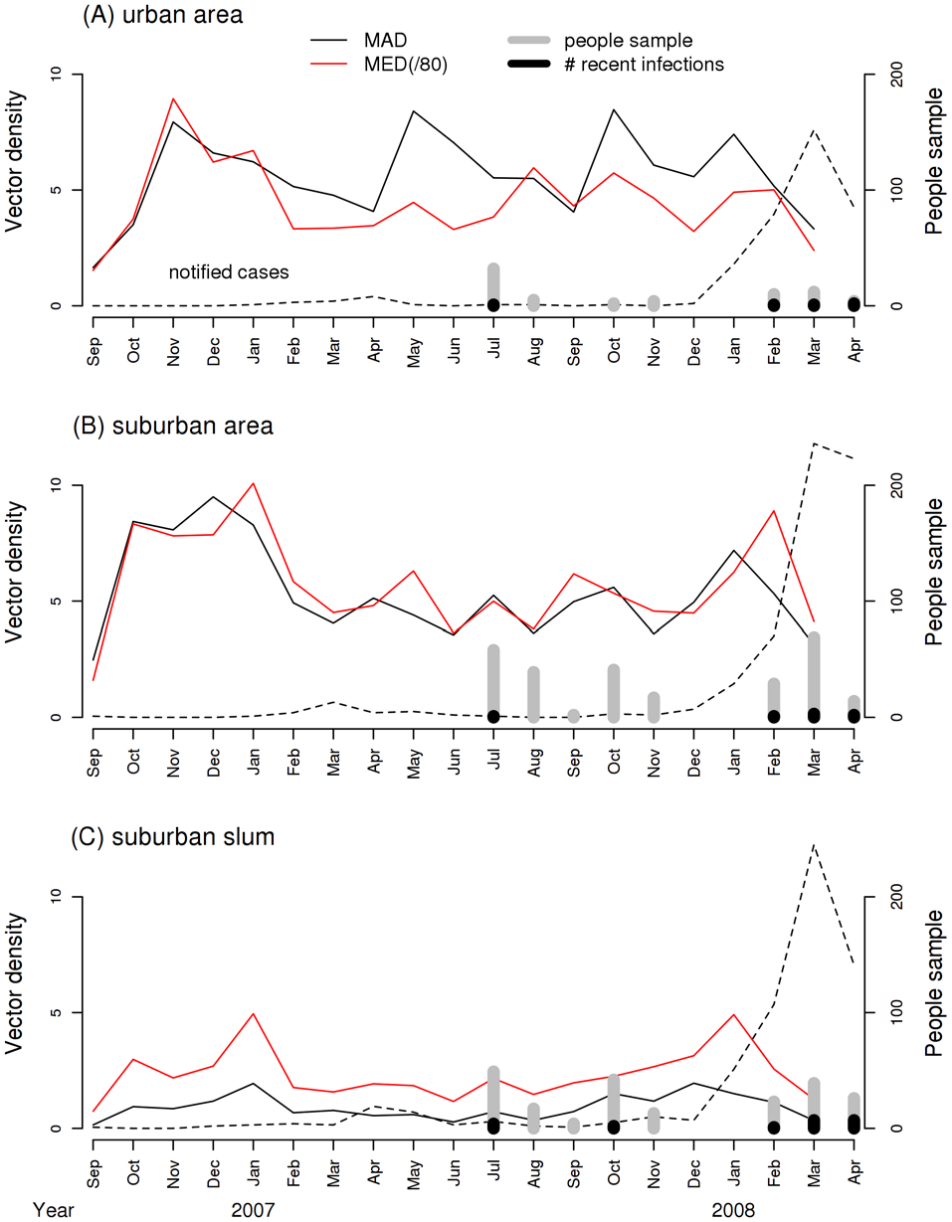
Time series of notified dengue cases, number of recent dengue infections and *Aedes aegypti* abundance. Time series of notified dengue cases in the three neighborhoods, according to the Healthy Authorities from the city of Rio de Janeiro (SMS-RJ 2008), number of serum samples collected, number of recent dengue infections, and *Ae. aegypti* abundance measured as mean adult density (MAD) and mean egg density (MED) in Higienópolis (urban), Tubiacanga (suburban) and Palmares (suburban slum), Rio de Janeiro, Brazil.

All administrative areas containing the studied neighborhoods had a history of dengue cases recorded by the local public health authorities [42]. Figure 3 shows the time series of reported dengue cases from Public Health Office of Rio de Janeiro city, with a clear peak between December/2007 and April/2008, during the present study. In 2008, the attack rates were: 45.94/‰ in Higienópolis, 35.17 in Galeão area (where the neighborhood of Tubiacanga is located) and 19.68 in Vargem Pequena area (where the suburban slum of Palmares is located). *Aedes aegypti* abundance was consistently high throughout the year in the urban and suburban sites (Higienópolis and Tubiacanga), and low in the suburban slum (Palmares). The largest increase in notified dengue fever cases began in December/2007 and apparently was not preceded by an increase in vector density as measured by our study. The mosquito indices (MAD and MED) time series fluctuated over the time. An increase in summer is clear in both suburban areas, but not in the urban area. The bars at the bottom of the picture, showing the number of recent dengue infections relative to the number of collected blood samples, coincide with the high peak of the 2008 epidemic. The Breteau index ranged from 4.20 to 11.32 in Higienópolis, 4.10 to 20.51 in Tubiacanga and 3.30 to 15.38 in Palmares.

### Serological surveys

Table 1 shows the results of the serological surveys. From 337 individuals, 247 provided paired serum samples (73.3%) (Higienópolis: paired/unpaired  = 28/16; Tubiacanga  = 117/45; Palmares  = 102/29). Age of participants ranged from 1 to 79 years, with an average of 16.9. There were 156 (46.3%) males and 181(53.7%) females. For education level, 29 (8.6%) were illiterate, 241 (71.5%) reported elementary school, 56 (16.6%) high school, and 11 (3.3%) college. Only 6.2% of the study subjects reported vaccination against yellow fever and 16% reported a previous history of dengue.

### Serology and RT-PCR

The combination of four methods provided diagnostic confirmation of dengue infection as follows: previous exposure to dengue (IgG) in the first survey detected in 199 (61.0%) out of the 326 individuals. Recent dengue infection (IgM) was detected in 30 individuals (4 in Higienópolis, 7 in Tubiacanga, and 19 in Palmares), which were subjected to nested RT-PCR and real-time RT-PCR (Table 1). DENV-RNA was detected in 5 individuals (4 DENV-2 and 1 DENV-3), by Nested RT-PCR and Real Time RT-PCR (TaqMan). Adopting quantitative real-time RT-PCR, we examined levels of DENV-RNA. The results revealed low viral RNA, ranging from 1 to 45 RNA copies/mL.

#### Recent dengue infection

From the first to the second survey, IgM seropositivity increased from 2.3% to 13.8% in Higienópolis (urban neighborhood), 1.3% to 4.1% in Tubiacanga (suburban neighborhood) and from 4.7% to 14.1% in Palmares (suburban slum) (Table 1). In Higienópolis, two out of 4 recent dengue infections were children (≤10 years); 3/4 was asymptomatic. In Tubiacanga, two out of 7 recent dengue infections occurred in children (≤10 years), 2/7 in 11–13 years old and 3/7 in adults (≥30 years); 6/7 individuals were asymptomatic. In Palmares, in 19 recent dengue infections, 9 (47.4%) were children (≤10 years), 6 (31.6%) were 11–18 years old and 4 (21.0%) were adults (≥25 years); 14/19 individuals had asymptomatic infections. Dengue seroconversion paired samples varied among the studies areas from 3.4% to 10.8% for IgM and 3.4% to 7.8% for IgG (Table 1). By combining data on IgM and IgG positivity in both serum samples, 9 (30.0%) individuals were classified as being primary infections, 19 (63.3%) as secondary infections, and 2 (6.7%) as inconclusive.

### Seroprevalence

**Figure 4:**
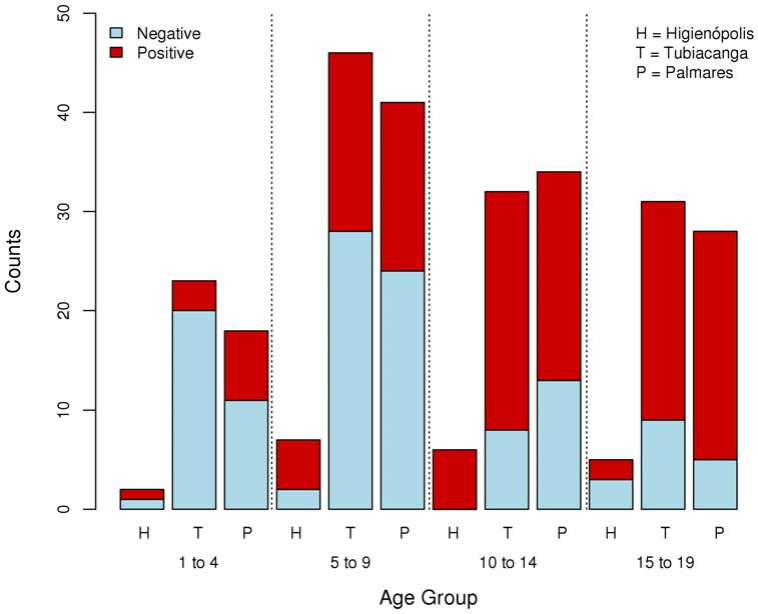
Dengue seroprevalence per age group. Dengue seroprevalence per age group (1 to 4, 5 to 9, 10 to 14, 15 to 19), red are positive and blue indicate negative cases in Higienópolis (urban), Tubiacanga (suburban) and Palmares (suburban slum) neighborhoods in Rio de Janeiro, Brazil.

**Figure 5:**
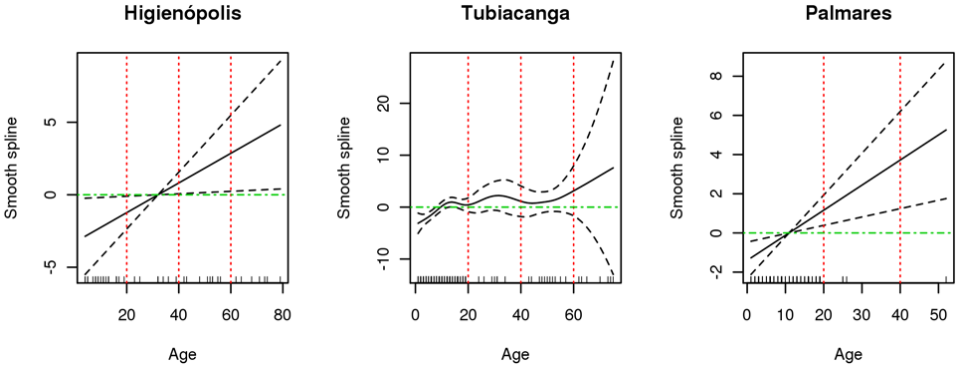
Effect of age on dengue seroprevalence. A smooth term estimating the effect of age on dengue seroprevalence is depicted with the black line. Dashed lines are the 95% confidence interval. Green line indicates no effect and red dotted lines divide age in 10 years interval in each studied neighborhood.

Dengue seroprevalence varied between the study areas. The age standardized proportions were 60.26% in Higienópolis, 56.07% in Tubiacanga and 77.44% in Palmares (Table 1, Fig. 4). In Higienópolis, the urban area, participation in the study was the lowest in all age groups, and the largest number of samples was in the interval of 5 to 9 years old. Frequency of seropositive samples increased with age (Fig. 4). In Tubiacanga a non-linear relationship between age and seroprevalence was observed, with a plateau at about 15 year old (Fig. 5). In the other two areas, the relationship between seroprevalence and age was linear and significant. Due to the non-linearity observed in Tubiacanga, we categorized the variable age, using cut points at 10 and 20 years old, to analyze the effect of age on seroprevalence in the multivariate models. The variable sex was significant only in Tubiacanga, while self-reported past dengue was a predictor of seropositivity in Tubiacanga and Palmares. Yellow fever vaccination was not statistically associated with dengue seropositivity in any study area (Table 2).

**Table 2:** Individual risk factors odds ratio for seroprevalence in three areas, Rio de Janeiro, Brazil.

Risk factor		Higienópolis			Tubiacanga			Palmares		
		N	OR	*P*	N	OR	*P*	N	OR	*P*
Sex	Male	22	1		73	1		61	1	
	Female	22	1.179e+08	0.99	89	1.93	0.05	70	0.66	0.26
Age	0–10 years	10	1		77	1		64	1	
	11–20 years	10	1.000e+00	1.00	55	7.91	0.001	60	3.42	0.01
	21–100 years	24	3.662e+08	0.99	30	25.39	0.001	7	4.15	0.22
Self-reported past history of dengue	No	34	1		143	1		106	1	
	Yes	10	2.478e+07	0.99	19	5.00	0.05	25	5.35	0.05
Yellow fever vaccination	No	40	1		147	1		129	1	
	Yes	4	7735420.4	0.99	15	1.96	0.27	2	1.634e+06	0.98

**Figure 6:**
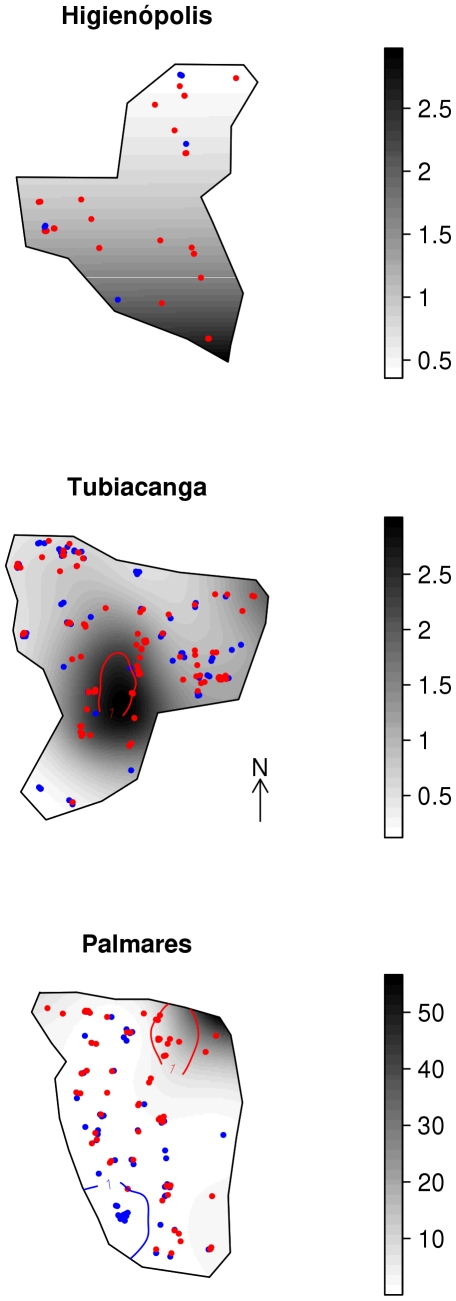
Spatial odds ratio for seroprevalence. Crude odds ratio surface estimated by a GAM model. Seropositive cases are represented in red dots and seronegative in blue. Red lines circle areas with significant higher positiveness density, whilst blue lines are significant lower prevalence levels in Higienópolis (urban), Tubiacanga (suburban) and Palmares (suburban slum) neighborhoods in Rio de Janeiro, Brazil.

Prevalence smooth maps, with darker gray colors indicating higher odds ratio (OR), are shown in Figure 6. In Higienópolis, the urban area, the spatial distribution of seroprevalence showed a linear North-South trend, with the highest odds ratios three times larger than the average value. However, no location in this area presented statistically significant differences in OR. Tubiacanga, the suburban area, presented similar variation in spatial odds ratio, with a high OR 3.0 region in the middle of the map, and this variation in chance significant (depicted by the red line in the map). In Palmares, the suburban slum, we observed the highest differences in seroprevalence distribution, with significantly high risk patch with OR  = 56 on the Northeast, where the main access to the community is located. Towards the South, a protective spatial effect is evident, and an area with a protective effect was observed, located close to a forested area. The OR maps resulting from the models adjusting for individual covariates (sex and age) presented a very similar pattern, and therefore are not shown.

### Spatial distribution of mosquito density and recent dengue infection

**Figure 7:**
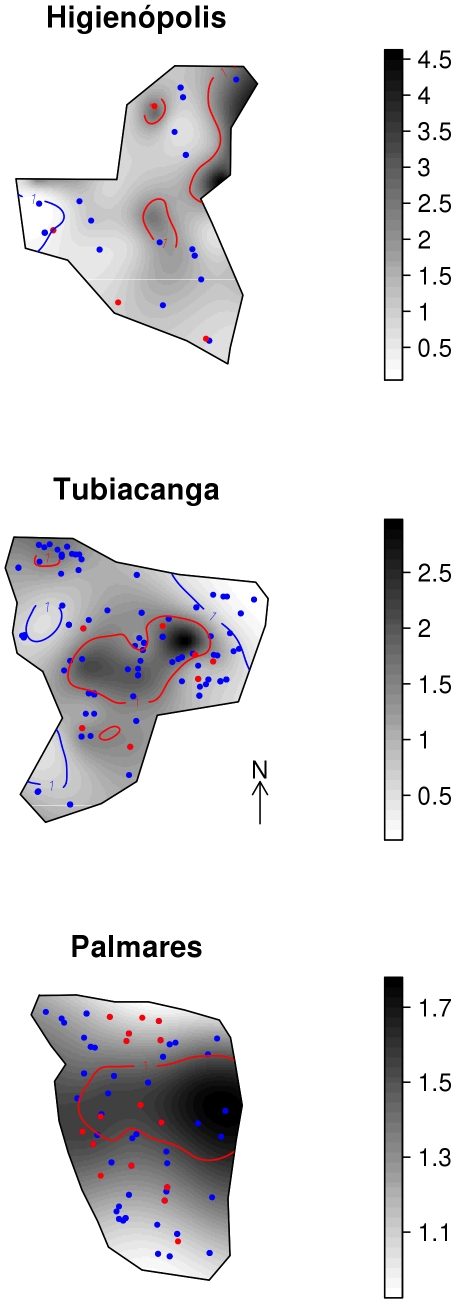
Map of adult *Aedes aegypti* distribution and recent dengue infections. Spatial relative risk of *Ae. aegypti* adults density in Higienópolis (urban), Tubiacanga (suburban) and Palmares (suburban slum) neighborhoods in Rio de Janeiro, Brazil. Red dots indicate the households of individuals with recent dengue infection, blue dots individuals with no evidence of recent dengue infection.

Figure 7 shows maps of adult *Ae. aegypti* abundance. Dots indicate the location of surveyed households with and without cases of recent dengue infection. Darker shades of gray indicate higher levels of mosquito abundance, measured in terms of relative risk (RR). Visual inspection, the only possible analysis due to the small number of recent dengue infections, suggests no evidence of a coincident pattern. In the urban area, Higienópolis, mosquito RR varied from ca 0.25 to 4.5, with a significantly high mosquito density area (depicted in red in the map). Only one of the four new infections is located inside or close to this area. In the suburban area, Tubiacanga, spatial variability in mosquito density was smaller, with RR going up to 3. Recent dengue infections are spread evenly over the entire area, just two in seven located inside a mosquito hotspot. Palmares, the suburban slum, showed the smallest variation in the vector density – with mosquitoes homogeneously covering the whole area, and recent dengue infections are also homogeneously distributed over the region, without any detectable pattern.

## Discussion

High dengue virus activity in Brazil during the past 20 years is evidenced by the large number of reported cases, in almost all states [13],[21],[22]. Rio de Janeiro, located in the Southeast Region of Brazil, is one of the most densely populated cities and has always been an important entry point for dengue viruses into the country [13],[43],[44] (Fig. 3).

In 2008, DENV-2 was the predominant serotype [12],[42]. In the current study, we confirmed the co-circulation of DENV-2 and DENV-3 serotypes in 5 individuals (4 DENV-2 and 1 DENV-3), by molecular methods, DENV-2 serotype invaded Rio de Janeiro 19 years before this study [10], when it caused an epidemic that resulted in about 100.000 notified cases. The 2008 DENV-2 epidemic struck a population were most children had no previous contact with this serotype, while most in the 10–20 years old group probably had experienced previous infections with either DENV-2 or DENV-3. Our results confirm this epidemiological scenario, with a high predominance of recent infections in children under 15 years old (18/30).

Although the number of recent dengue infections was small, we decided to present the data because it is rare to have any recent infection data in population surveys. The epidemic that occurred during our field work presented the largest number of severe cases in children [12],[42]. However, in our data, only 23.3% of infections were symptomatic, suggesting that even during such severe epidemic, silent circulation of the virus is highly prevalent [20],[21],[45],[46]. A consequence of high frequency of asymptomatic infections is that measures of notified cases greatly underestimate the true incidence of infection and difficult the identification of high risk transmission areas within cities [47].

We observed events of recent dengue infection in residences located in areas with low mosquito densities, suggesting that infection took place out of the residence, either in other premises – school, for instance – or outdoors, (where children in these neighborhoods stay most of daytime, when *Aedes* mosquitoes are more active). However, the lack of coherence between household mosquito counts and recent dengue infection should be further investigated in future work, by comparing the current data with infected *Ae. aegypti* information [48]. In parallel, information on human population movement patterns could also bring further insight on dengue fever transmission dynamics and the main places of transmission, eventually serving to build an early warning system for dengue outbreaks.

Entomological surveillance is of great importance for early detection of transmission risk and for directing vector control measures. However, in Brazil, vector surveillance using Premise and Breteau indices correlates poorly with dengue incidence [49],[50],[51], and moderately with the rate of epidemic growth [25]. In Puerto Rico a study [52] to investigate the relationship between serological and epidemiological surveys and mosquito density showed that none of the household characteristics evaluated was significantly associated with recent dengue infection, except the number of female *Ae. aegypti* per person. In Colombia, the only entomological factor related to dengue infection in humans was the pooled infection rate of mosquitoes. It would be helpful to discover the threshold of mosquito density that would trigger an epidemic [51],[53].

Epidemiological studies have identified statistical risk factors for human infection or diseases [54],[55],[56]. Statistical models can bridge the gaps between landscape ecology, vector biology and human epidemiology, providing a sound approach to understanding risk and planning for control in heterogeneous environments, especially when the models are based on the ecology of the local vector populations [55]–[58]. Additionally, understanding the space and time distribution of risk for mosquito-borne infections is an important step in planning and implementing effective infection control measures [59],[60]. This is because space and time are two important dimensions in describing epidemic dynamics and risk distribution [61].

Our results point to larger spatial heterogeneity in dengue seroprevalence in the most isolated areas – Tubiacanga and Palmares. In Tubiacanga, seroprevalence concentrated in the area with more intense commercial activity, schools and the main bus station. In Palmares, seroprevalence was concentrated in the slum entrance, also an area of high commercial activity and human movement. We hypothesize that such isolated populations are too small to maintain the dengue virus endemically and that the observed seroprevalence maps are the result of multiple viral introductions through the last 20 years, always through the same entrance. Such spatial clustering of dengue has being reported in the literature [45],[46], and supports the hypothesis that mosquito-borne disease incidence is highly focal [46],[62]. On the other hand, a spatial pattern was not observed in Higienópolis, a neighborhood with multiple accesses and surrounded by slums with high population density.

These results highlight the important role on dengue transmission, of public spaces where human movement is intense, possibly more important than the households. Further characterization of human movement patterns should provide additional information in the understanding of dengue transmission dynamics [63]. Some authors have suggested that people rather than mosquitoes rapidly move dengue virus within and among communities [64],[65]. The present study is consistent with this information.

Our results must be considered in the context of the limitations of the serological survey. First, the small number of recent dengue infections precluded a more adequate modeling of incidence versus mosquito density associations. Second, the age distribution, particularly in Higienópolis, was not comparable to the other areas. Third, households in the entomological and serological surveys did not match exactly what may precluded the identification of association between mosquito abundance and risk of infection.

This study contributes to a better understanding of the dynamics of dengue in Rio de Janeiro by assessing the relationship between dengue seroprevalence, recent dengue infection, and vector density. In conclusion, the variation in spatial seroprevalence patterns inside the neighborhoods, with significantly higher risk patches close to the areas with the greatest human movement, suggests that humans may be responsible for virus inflow to small neighborhoods in Rio de Janeiro. Surveillance guidelines should be further discussed, considering these findings, specially the spatial patterns for both human and mosquito populations.

## Supporting Information

Checklist S1 STROBE checklist
(0.07 MB DOC)
