# Supplementary material for: Spatial Evaluation and Modeling of Dengue Seroprevalence and Vector Density in Rio de Janeiro, Brazil
Source: PLoS Negl Trop Dis. 2009 Nov 10;3(11):e545. doi: 10.1371/journal.pntd.0000545 (PMC2768822; doi:10.1371/journal.pntd.0000545)
Supplement: Checklist S1 — STROBE checklist (0.07 MB DOC) [file pntd.0000545.s001.doc]

STROBE Statement—Checklist of items that should be included in reports of ***cross-sectional studies***

|  | Item No | Recommendation |
| --- | --- | --- |
| **Title and abstract** | 1 | (*a*) **OK** |
|  |  | (*b*) **OK** |
| Introduction | | |
| Background/rationale | 2 | **OK** |
| Objectives | 3 | **OK, page 6** |
| Methods | | |
| Study design | 4 | **Pages 8,9** |
| Setting | 5 | **Page 7, 8** |
| Participants | 6 | (*a*) **Pages 8,9,11** |
| Variables | 7 | Outcomes – **page 9**  exposures, predictors, potential confounders, and effect modifiers – **pages 7,8**  diagnostic criteria – **pages 8, 9** |
| Data sources/ measurement | 8* | For each variable of interest, give sources of data and details of methods of assessment (measurement). Describe comparability of assessment methods if there is more than one group – **pages 9 to 13** |
| Bias | 9 | Describe any efforts to address potential sources of bias – **page 8** |
| Study size | 10 | Explain how the study size was arrived at – **page 8** |
| Quantitative variables | 11 | Explain how quantitative variables were handled in the analyses. If applicable, describe which groupings were chosen and why **– pages 12, 13** |
| Statistical methods | 12 | (*a*) Describe all statistical methods, including those used to control for confounding – **pages 12, 13** |
|  |  | (*b*) Describe any methods used to examine subgroups and interactions – **comparison of areas all through the results section** |
|  |  | (*c*) Explain how missing data were addressed – **No missing variables, only some individuals had only one sample collected. Page 9** |
|  |  | (*d*) If applicable, describe analytical methods taking account of sampling strategy- **NO** |
|  |  | (*e*) Describe any sensitivity analyses – **not done**. |
| Results | | |
| Participants | 13* | (a) Report numbers of individuals at each stage of study—eg numbers potentially eligible, examined for eligibility, confirmed eligible, included in the study, completing follow-up, and analysed – **page 14, table 1** |
|  |  | (b) Give reasons for non-participation at each stage – **pages 8,9** |
|  |  | (c) Consider use of a flow diagram – **Not done, as there are too many figures already.** |
| Descriptive data | 14* | (a) Give characteristics of study participants (eg demographic, clinical, social) and information on exposures and potential confounders – **Table 1** |
|  |  | (b) Indicate number of participants with missing data for each variable of interest – **missing data is related only to one blood sample not taken (see item 12.c)** |
| Outcome data | 15* | Report numbers of outcome events or summary measures – **Table 1** |
| Main results | 16 | (*a*) Give unadjusted estimates and, if applicable, confounder-adjusted estimates and their precision (eg, 95% confidence interval). Make clear which confounders were adjusted for and why they were included – **Table 2 and OR Maps** |
|  |  | (*b*) Report category boundaries when continuous variables were categorized – **Figure 4, page 16** |
|  |  | (*c*) If relevant, consider translating estimates of relative risk into absolute risk for a meaningful time period – **NO relevant** |
| Other analyses | 17 | Report other analyses done—eg analyses of subgroups and interactions, and sensitivity analyses – **OR maps discussed as a main result. Figures 6,7** |
| Discussion | | |
| Key results | 18 | Summarise key results with reference to study objectives – **page 18, 19,20** |
| Limitations | 19 | Discuss limitations of the study, taking into account sources of potential bias or imprecision. Discuss both direction and magnitude of any potential bias – **page 21** |
| Interpretation | 20 | Give a cautious overall interpretation of results considering objectives, limitations, multiplicity of analyses, results from similar studies, and other relevant evidence – **page 21** |
| Generalisability | 21 | Discuss the generalisability (external validity) of the study results – **page 21** |
| Other information | | |
| Funding | 22 | Give the source of funding and the role of the funders for the present study and, if applicable, for the original study on which the present article is based - **OK** |

*Give information separately for exposed and unexposed groups.

**Note:** An Explanation and Elaboration article discusses each checklist item and gives methodological background and published examples of transparent reporting. The STROBE checklist is best used in conjunction with this article (freely available on the Web sites of PLoS Medicine at http://www.plosmedicine.org/, Annals of Internal Medicine at http://www.annals.org/, and Epidemiology at http://www.epidem.com/). Information on the STROBE Initiative is available at www.strobe-statement.org.
